# Supplementary material for: Perceptual sensitivity is explained by optimization for ecological tasks
Source: bioRxiv. 2026 Oct 5:2026.09.28.755158. Preprint. [Version 1] doi: 10.64898/2026.09.28.755158 (PMC13691993; doi:10.64898/2026.09.28.755158)
Supplement: Supplementary file 1 [file NIHPP2026.09.28.755158V1-supplement-1.pdf]

bioRxiv preprint doi: https://doi.org/10.64898/2026.09.28.755158; this version posted October 5, 2026. The copyright holder for this preprint (which was not certified by peer review) is the author/funder, who has granted bioRxiv a license to display the preprint in perpetuity. It is made available under a CC-BY 4.0 International license.

SUPPLEMENTARY INFORMATION

Perceptual sensitivity is explained by optimization for ecological tasks

Mark R. Saddler, Torsten Dau, Josh H. McDermott

TABLE OF CONTENTS

Supplementary Figure 1. Deterministic model ecological training task performance

Supplementary Figure 2. Additional modulation masking experiments

Supplementary Figure 3. Additional binaural masking level difference experiments

Supplementary Figure 4. Nonsimultaneous masking experiments

Supplementary Figure 5. Simultaneous masking experiments

Supplementary Figure 6. Remaining psychoacoustic experiments

bioRxiv preprint doi: https://doi.org/10.64898/2026.09.28.755158; this version posted October 5, 2026. The copyright holder for this preprint (which was not certified by peer review) is the author/funder, who has granted bioRxiv a license to display the preprint in perpetuity. It is made available under a CC-BY 4.0 International license.

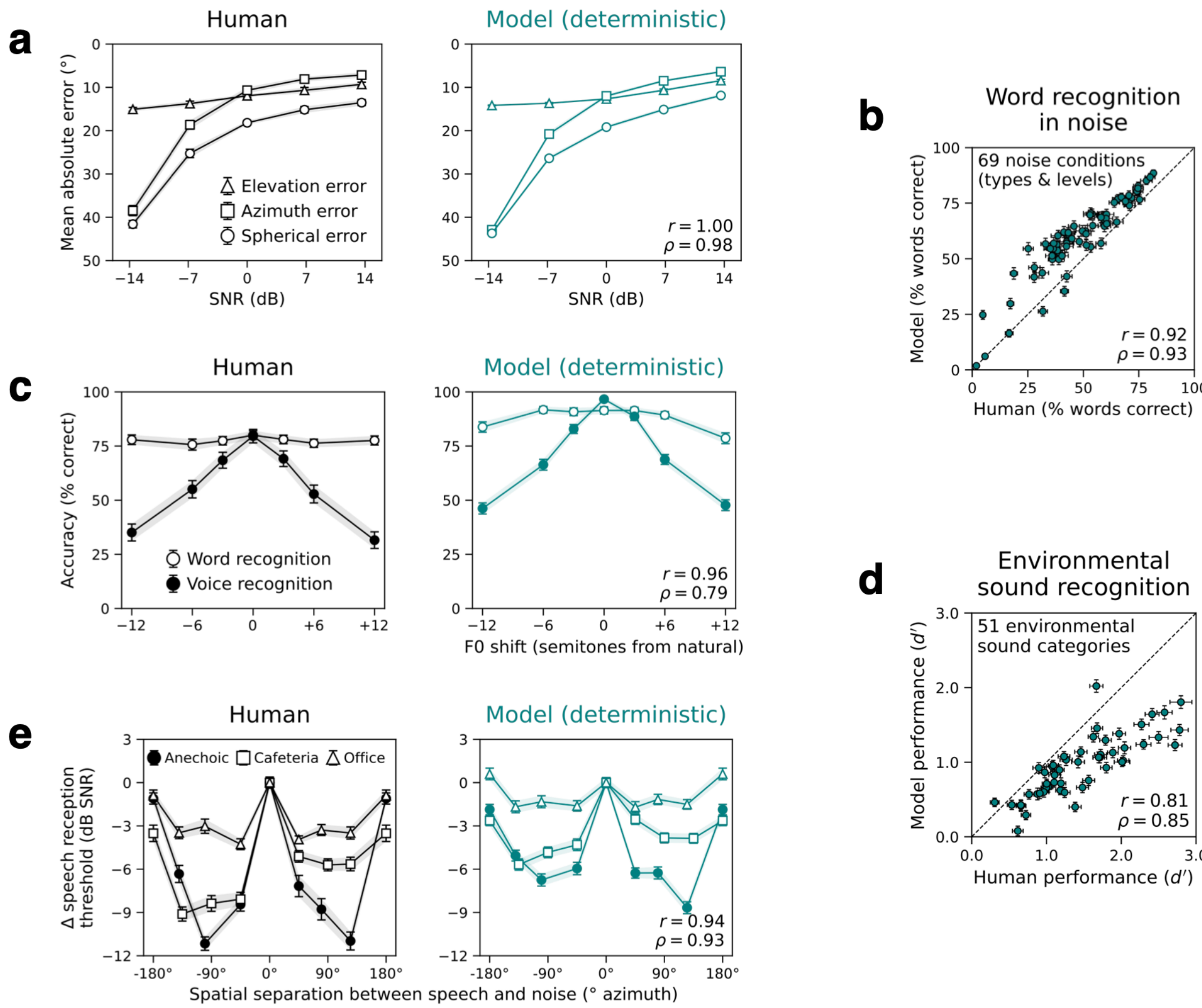

Supplementary Figure 1. The fully deterministic model optimized for ecological hearing tasks exhibits human-level sound localization and recognition task performance. (**a**) Comparison of human and model localization performance, assessed by measuring mean absolute azimuth, elevation, and spherical error as a function of signal-to-noise ratio. (**b**) Comparison of human and model word recognition accuracy across 69 background noise conditions. (**c**) Human and model word and voice recognition accuracy measured using speech from familiar talkers whose voice has been F0-shifted. (**d**) Comparison of human and model environmental sound recognition performance across 51 different sound classes. (**e**) Human and model speech reception thresholds, measured as a function of the spatial separation between speech and noise in three acoustically distinct rooms. Human-model similarity for each task is quantified using Pearson (r) and Spearman (ρ) correlation coefficients. Human data in (**a**), (**b**), and (**c**) are from ref.[17]. Human data in (**d**) and (**e**) are from refs.[25,27], respectively. Error bars indicate ±1 s.e.m.

bioRxiv preprint doi: https://doi.org/10.64898/2026.09.28.755158; this version posted October 5, 2026. The copyright holder for this preprint (which was not certified by peer review) is the author/funder, who has granted bioRxiv a license to display the preprint in perpetuity. It is made available under a CC-BY 4.0 International license.

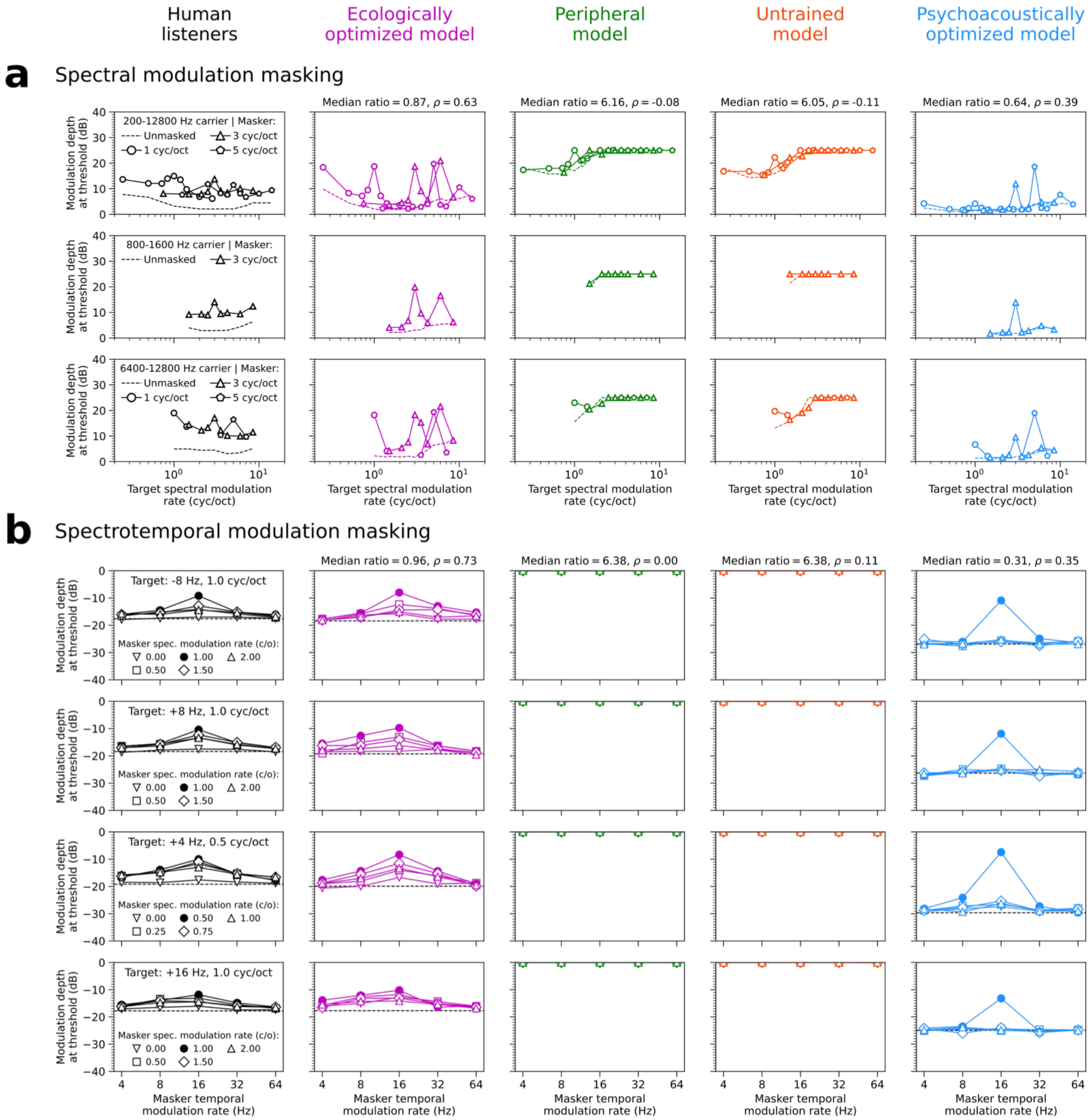

Supplementary Figure 2. Additional modulation masking experiments. (**a**) Spectral modulation detection thresholds measured in the presence of different masker spectral modulations (different lines) imposed on different carrier noise bands (different rows). (**b**) Spectrotemporal modulation detection thresholds measured for different combinations of target and masker modulations. Rows correspond to four different target spectrotemporal modulations. Masker spectrotemporal modulations are specified along the x-axis (temporal modulation rates) and the different lines (spectral modulation rates).

bioRxiv preprint doi: https://doi.org/10.64898/2026.09.28.755158; this version posted October 5, 2026. The copyright holder for this preprint (which was not certified by peer review) is the author/funder, who has granted bioRxiv a license to display the preprint in perpetuity. It is made available under a CC-BY 4.0 International license.

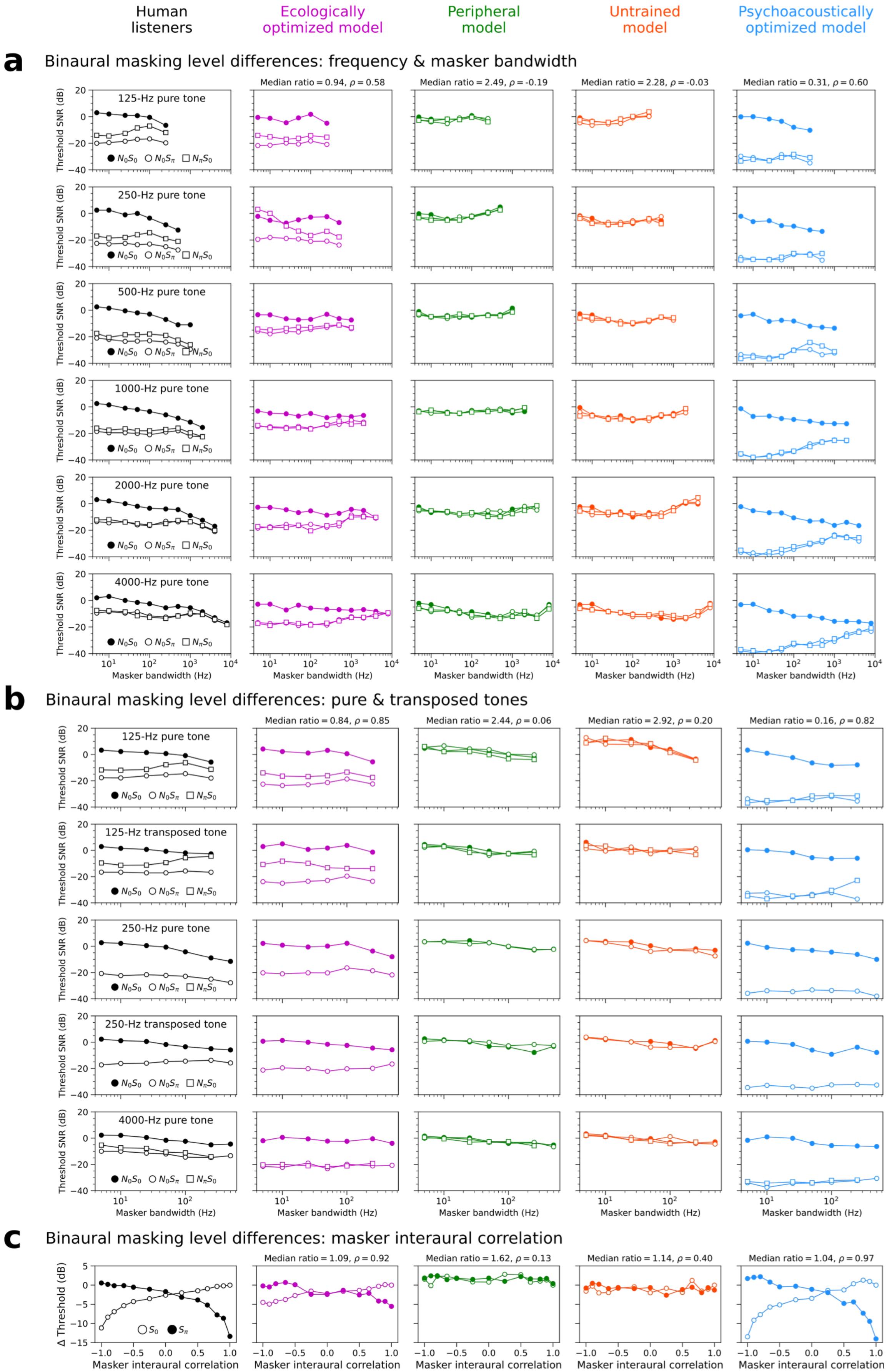

bioRxiv preprint doi: https://doi.org/10.64898/2026.09.28.755158; this version posted October 5, 2026. The copyright holder for this preprint (which was not certified by peer review) is the author/funder, who has granted bioRxiv a license to display the preprint in perpetuity. It is made available under a CC-BY 4.0 International license.

Supplementary Figure 3. Additional binaural masking level difference experiments. (**a**) Binaural tone-in-noise detection thresholds measured as a function of pure tone frequency, masker bandwidth, and interaural phase condition. The interaural phase of the noise (N) and signal (S) are each denoted with subscripts: 0 = in phase between the ears, π = 180° out of phase. (**b**) Analogous thresholds measured for pure tones and transposed tones with 4000 Hz carrier frequencies. (**c**) Binaural masking level differences (BMLDs) measured as a function of masker interaural correlation. BMLDs indicate the change in detection threshold relative to the S_0 condition with a masker interaural correlation of one. Median model-to-human threshold ratios and human-model Spearman correlation coefficients (ρ) are computed across the stimulus conditions within each experiment.

bioRxiv preprint doi: https://doi.org/10.64898/2026.09.28.755158; this version posted October 5, 2026. The copyright holder for this preprint (which was not certified by peer review) is the author/funder, who has granted bioRxiv a license to display the preprint in perpetuity. It is made available under a CC-BY 4.0 International license.

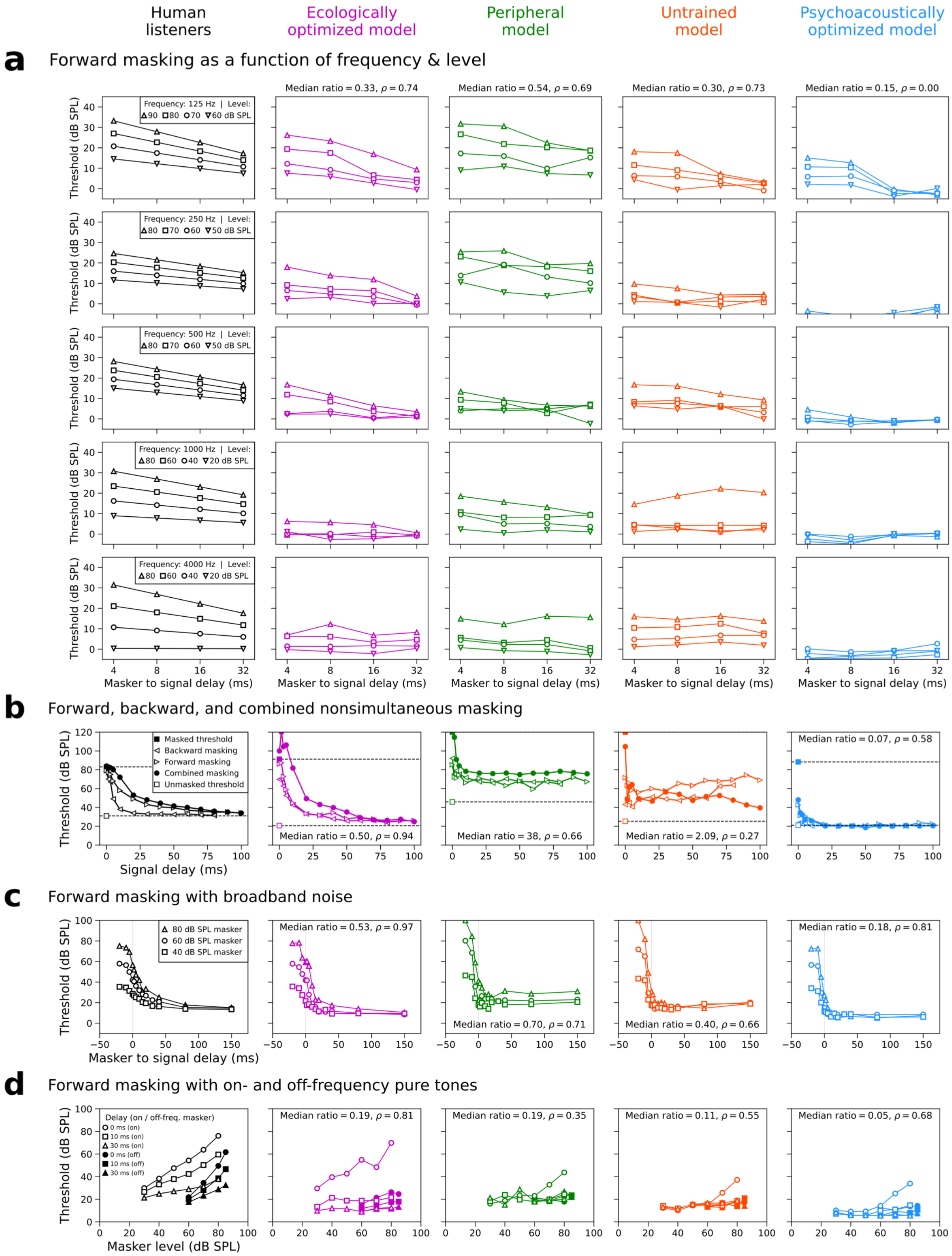

Supplementary Figure 4. Nonsimultaneous masking experiments. (**a**) Forward-masked pure tone detection thresholds measured as a function of frequency (different rows), masker levels (different lines), and the temporal separation between the masker offset and the signal onset (x-axis). (**b**) Detection thresholds for a 6000-Hz pure tone pip as a function of

bioRxiv preprint doi: https://doi.org/10.64898/2026.09.28.755158; this version posted October 5, 2026. The copyright holder for this preprint (which was not certified by peer review) is the author/funder, who has granted bioRxiv a license to display the preprint in perpetuity. It is made available under a CC-BY 4.0 International license.

the temporal separation between signal and masker. In the backward masking condition, the masker occurs after the signal. In the forward masking condition, the masker occurs before the signal. In the combined masking condition, maskers occur both before and after the signal. (**c**) Forward-masked detection thresholds for a 4000-Hz pure tone as a function of masker-to-signal delay for three different levels of broadband noise masker. (**d**) Forward-masked detection thresholds for a 4000-Hz pure tone as a function of masker level and delay for on- and off-frequency pure tone maskers (4000 and 2400 Hz, respectively).

bioRxiv preprint doi: https://doi.org/10.64898/2026.09.28.755158; this version posted October 5, 2026. The copyright holder for this preprint (which was not certified by peer review) is the author/funder, who has granted bioRxiv a license to display the preprint in perpetuity. It is made available under a CC-BY 4.0 International license.

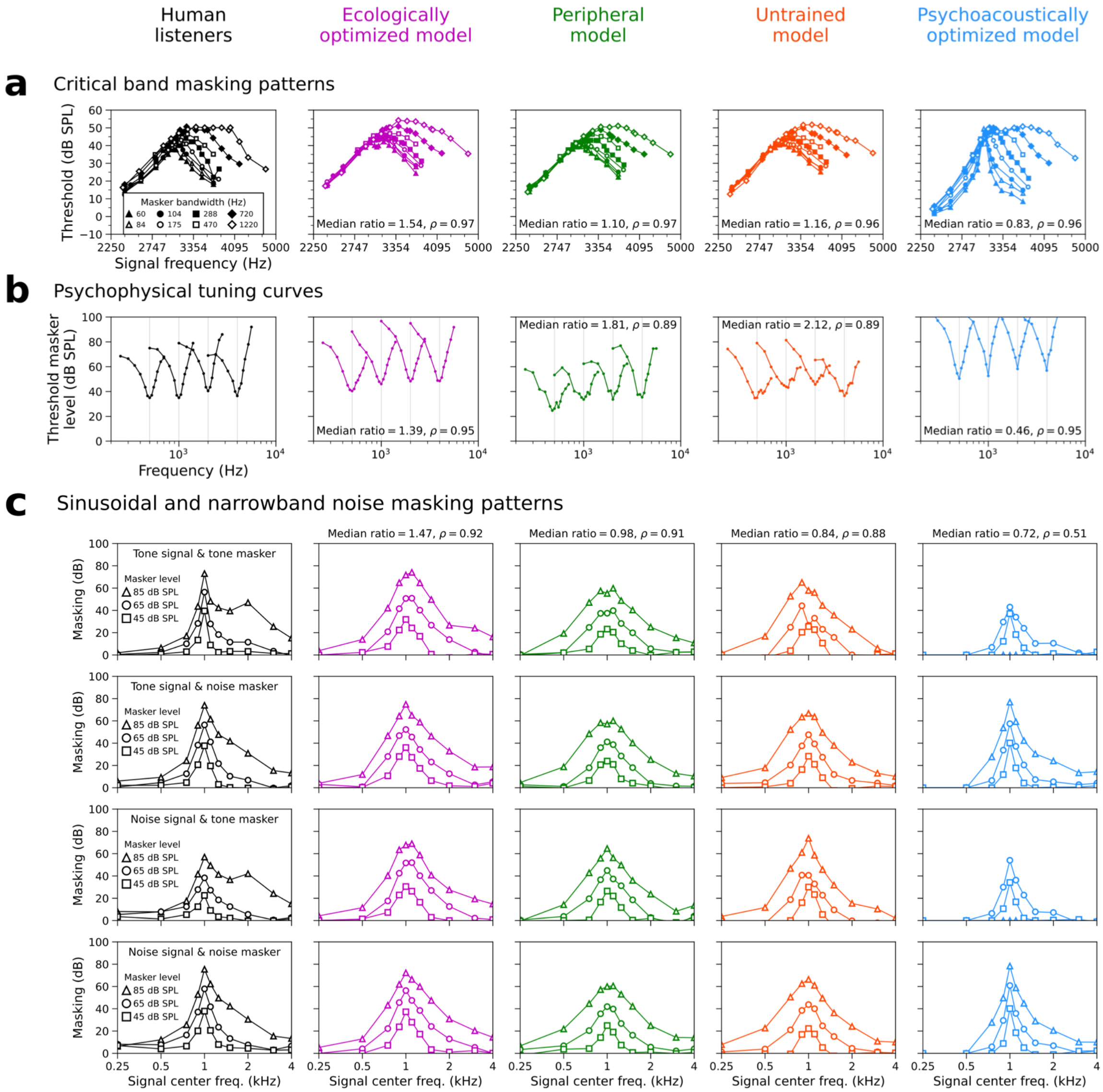

Supplementary Figure 5. Simultaneous masking experiments. (a) Pure tone detection thresholds measured in the presence of masking noise whose low frequency cutoff was fixed at 3030 Hz and whose bandwidth varied. (b) Psychophysical tuning curves show the level of narrowband noise needed mask a pure tone with frequency 500, 1000, 2000, or 4000 Hz. The narrowband noise was 25-Hz wide and centered at the frequency plotted on the x-axis. (c) Sinusoidal and narrowband noise masking patterns. In this masked detection experiment, the signal and the masker were each either a pure tone or an 80-Hz wide narrowband noise. The four possible signal-masker pairings are shown in the four rows. The masker was always centered at 1 kHz and signal detection thresholds were measured as a function of signal center frequency for three masker levels. The amount of masking was computed as the threshold increase relative to unmasked detection.

bioRxiv preprint doi: https://doi.org/10.64898/2026.09.28.755158; this version posted October 5, 2026. The copyright holder for this preprint (which was not certified by peer review) is the author/funder, who has granted bioRxiv a license to display the preprint in perpetuity. It is made available under a CC-BY 4.0 International license.

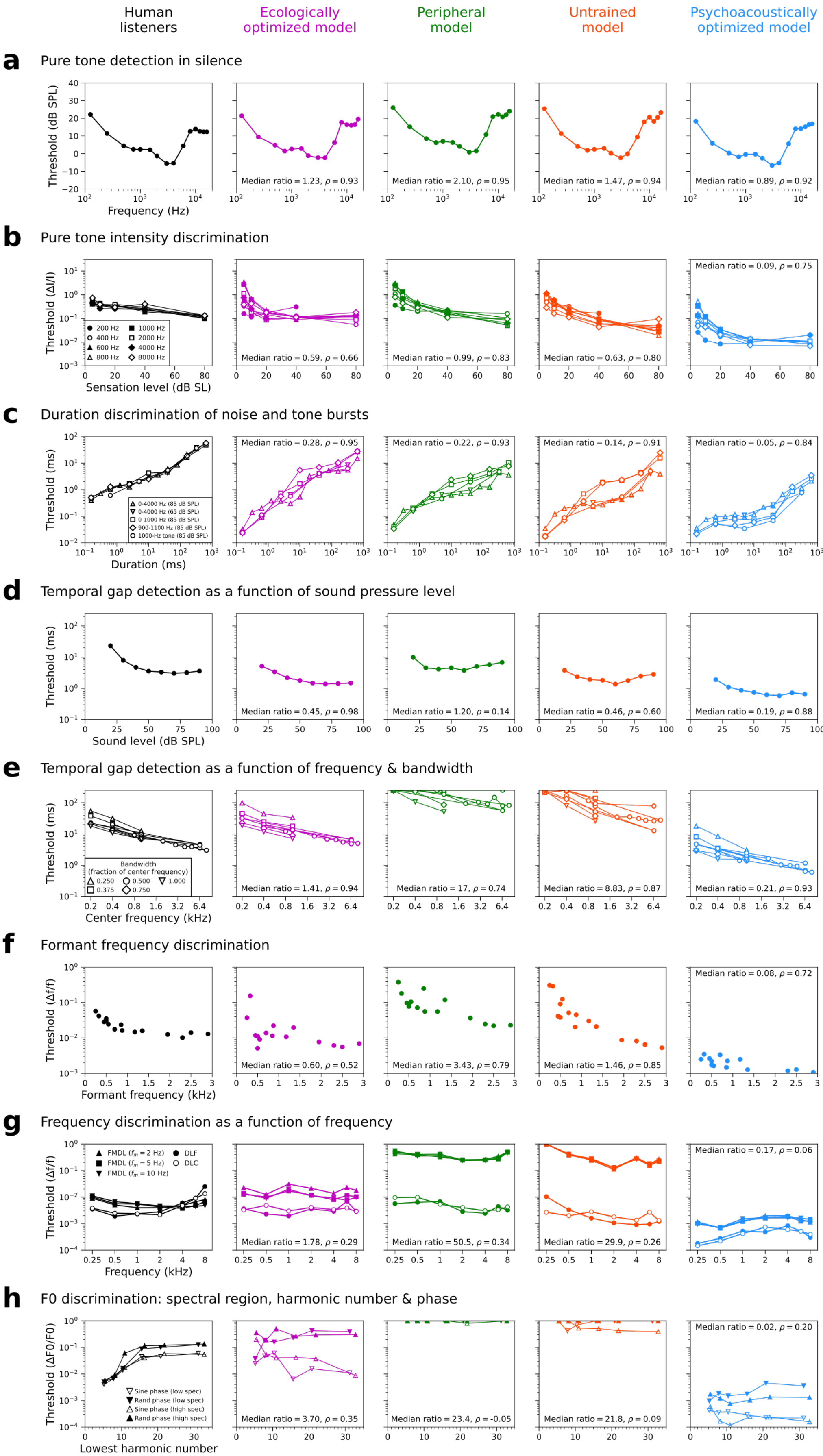

bioRxiv preprint doi: https://doi.org/10.64898/2026.09.28.755158; this version posted October 5, 2026. The copyright holder for this preprint (which was not certified by peer review) is the author/funder, who has granted bioRxiv a license to display the preprint in perpetuity. It is made available under a CC-BY 4.0 International license.

Supplementary Figure 6. Remaining psychoacoustic experiments. (**a**) Pure tone detection thresholds as a function of frequency. (**b**) Pure tone intensity discrimination thresholds as a function of sensation level. (**c**) Duration discrimination thresholds as a function of stimulus condition and duration. (**d**) Temporal gap detection thresholds for broadband noise as a function of sound level. (**e**) Temporal gap detection thresholds for narrowband noise as a function of center frequency and bandwidth. (**f**) Formant frequency discrimination thresholds for the first and second formants of ten vowels as a function of formant frequency. (**g**) Frequency discrimination thresholds measured as a function of frequency with three different tasks: frequency change detection (DLC), up/down frequency discrimination (DLF), and frequency modulation detection (FMDL). Frequency modulation detection thresholds were measured for three different frequency modulation rates. (**h**) Complex tone F0 discrimination thresholds as a function of spectral region, lowest harmonic number, and harmonic phase. The low (2.5 to 3.5 kHz) and high (5 to 7 kHz) spectral regions had fixed passbands, and the lowest harmonic number was varied by changing F0.
